# Therapeutic role of glutamine in management of radiation enteritis: a meta-analysis of 13 randomized controlled trials

**DOI:** 10.18632/oncotarget.15741

**Published:** 2017-02-25

**Authors:** De-dong Cao, Hui-lin Xu, Min Xu, Xiang-yun Qian, Zhu-cheng Yin, Wei Ge

**Affiliations:** ^1^ Department of Oncology, RenMin Hospital of WuHan University, WuHan, Hubei, P.R. China; ^2^ Department of Oncology, The Fifth Hospital of Wuhan, Wuhan, P.R. China

**Keywords:** radiation enteritis, glutamine, radiotherapy, meta-analysis, evidence based medicine

## Abstract

**Objective:**

To systematically evaluate the clinical efficacy of glutamine in treating radiation enteritis in cancer patients treated with radiotherapy.

**Methods:**

Electronic databases including Pubmed, Embase, the Cochrane library, and CNKI were systematically searched, until April 2016. Randomized controlled trials (RCT) of glutamine in the treatment of radiation enteritis in cancer patients were searched, and RevMan 5.3 software was used for Meta-analysis.

**Results:**

A total of 13 RCTs were included, involving 979 patients. The results of meta-analysis showed that the total efficacy of glutamine was higher for patients with radiation enteritis compared with that in control group, however, there was no statistically significant difference(OR = 3.07, 95%CI: 0.79-11.96; *P* > 0.05). The combined ORs for all 5 grades(from grade 0 to grade 4) of radiation enteritis in patients receiving glutamine were 2.06, 1.35, 0.55, 0.62 and 0.59, respectively(*P* > 0.05 for all). Glutamine also failed to significantly improve the symptoms of radiation enteritis in terms of tenesmus, abdominal cramping and blood in bowel movement(*P* > 0.05).

**Conclusions:**

Implementation of glutamine fails to improve the severity and symptoms in patients with radiation enteritis.

## INTRODUCTION

Radiotherapy has been used for treating cancer worldwide, nevertheless it is accompanied with adverse effects on the normal organs or tissues [1]. Gastrointestinal tract is a sensitive organ to radiotherapy, and radiation enteritis is one of the adverse effects caused by radiotherapy [2]. It refers to the abdominal, intestinal complications after pelvic or peritoneal radiation, and usually divided into acute and chronic enteritis. Acute radiation enteritis is a clinical term characterized with edema, inflammation, ulceration, epithelial degeneration, decrease in mitotic activity and crypt abscess in gastrointestinal tract [3, 4]. In clinical practice, acute radiation enteritis are commonly occurred after two weeks of radiation, and often cause a number of intestinal symptoms such as diarrhea, defecation pain and mucus, blood in the stool, and tenesmus [5]. In contrast to acute radiation enteritis, chronic radiation enteritis are developed from acute radiation enteritis or direct exposure to local tissue ischemia and long lasting interstitial fibrosis, vascular occlusive meningitis which are caused by chronic inflammation. It exhibited in recurrent blood in the stool, abdominal pain, diarrhea, even of intestinal stenosis, ulcers and fistula formation [6, 7].

Studies showed that the incidences of radiation enteritis ranged from 5% to 20% when using radiation therapy for patients with prostate cancer, colorectal cancer, bladder cancer, cervical cancer, testicular cancer, and uterine cancer [8, 9]. In cancer patients suffering from radiation enteritis, there is increasing need for development of effective and standard radio-protective regimens to recede or minimize the damage caused by radiation. Although the uses of steroids, antidiarrheal agents, amifostine, glutamine, and other agents are reported in studies and showed certain efficacy, currently there are no standard treatments for radiation enteritis [2, 9, 10].

Glutamine is one kind of neutral amino acid with the roles of providing energy source for intestinal endothelial cells and enhancing local bowel immune function [10]. The serum level of glutamine is usually decreased when patients are under stress conditions, such as radiotherapy. As a result, the recovery of the integrity of intestinal tract epithelium is further impaired [8]. Adding external glutamine for patients with radiation enteritis is believed to be beneficial, with the strong evidence provided by the animal studies [11, 12]. However, available clinical data failed to demonstrate the beneficial effect of glutamine during pelvic radiotherapy [2, 6, 10]. In contrary, in China, compound glutamine capsules showed positive effect in eliminating or minimizing incidence of radiation enteritis [3, 7, 13–15]. In this study, we aimed to evaluate the role of glutamine, which has controversial efficacy, for improvement and management of radiation enteritis.

## RESULTS

### Study selection and characteristics

**Figure 1:**
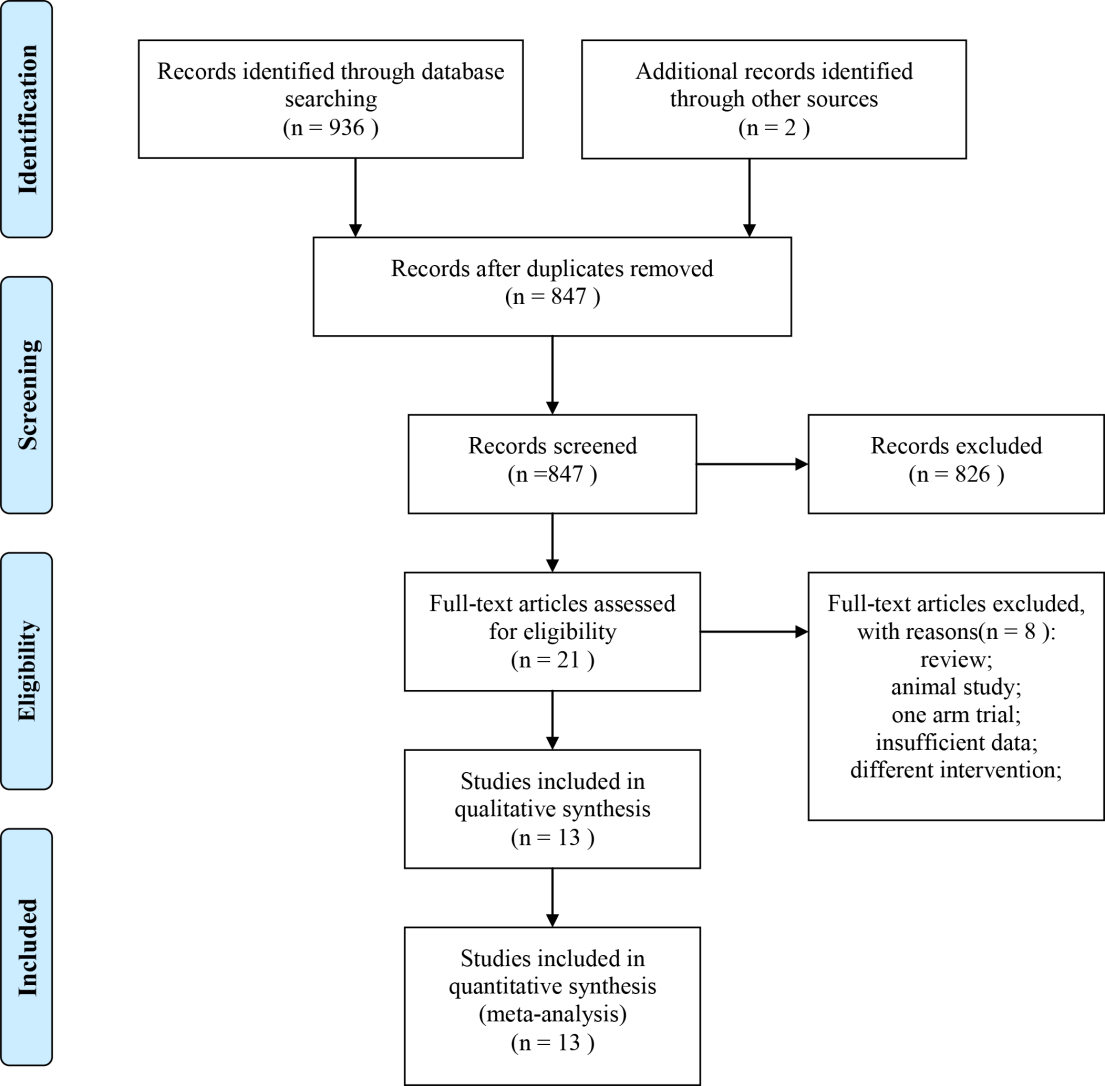
Flow chart of selection of eligible studies

A total of 938 references published between 1990 and 2016 were identified after initial search using combined search terms as described. 847 studies were selected after reviewing of abstracts and titles if they did not meet the criteria mentioned above. 21 potential eligible articles were identified for the following full-text review, with a perfect agreement(k=0.96). Upon further review, 5 articles were excluded as inadequate data and 3 studies were eliminated due to bias of publication or review article. Finally, 13 eligible trials [10, 13–24] were included for this meta-analysis(Figure 1). All studies reported the definition of radiation enteritis and criteria of efficacy for cancer patients receiving pelvic and/or abdominal radiotherapy. The total number of participants involved was 979, ranging from 60 to 129 cases each trial. The age of these patients ranged from 30 to 78 years. The characteristics of retained trials are presented in Table 1. Studies are listed twice if they showed data for both prevention and efficacy of glutamine for radiation enteritis.

**Table 1:** Baseline characteristics of included studies

Studies	N		Sex		Age		Interventions		Outcomes
	glutamine	control	Male	Female	glutamine	control	glutamine	control	
Kozelsky, 2003	64	65	88	41	69.5	69.0	4g, po, bid	placebo	severity of radiation enteritis; PRS; QoL
Yang, 2004	36	33	23	46	48.3±8.9	49.9±8.5	4#, po, tid	placebo	severity of radiation enteritis; PRS
Zhou, 2007	45	40	NA	NA	45.4±5.1	45.6±6.8	3#, po, tid	placebo	severity of radiation enteritis
Rao, 2009	45	35	49	31	30-68	30-68	3#, po, tid	placebo	severity of radiation enteritis; PRS
Qi, 2011	38	38	32	44	32-78	32-78	3#, po, tid	placebo	severity of radiation enteritis
Wu, 2011	32	30	28	34	33-67	33-67	4#, po, tid	placebo	severity of radiation enteritis; PRS; QoL
Zhang, 2012	30	30	0	60	32-61	34-63	4#, po, tid	placebo	severity of radiation enteritis
Gu, 2012	32	31	0	63	49	48.5	0.3g/Kg, iv, qd	placebo	severity of radiation enteritis
Zhao, 2013	42	36	30	48	31~75	32-78	4#, po, tid	placebo	severity of radiation enteritis
Vidal-Casariego, 2014	34	35	45	24	64.9±9.7	68.1±10.0	30g/d, po, qd	placebo	severity of radiation enteritis
Meng, 2014	22	20	20	22	51.2±3.3	50.8±2.9	2#, po, tid	placebo	severity of radiation enteritis
Chi, 2015	42	40	0	82	54.5±6.7	52.0±7.3	0.3g/Kg, iv, qd	placebo	severity of radiation enteritis
Chen, 2015	44	40	40	44	31-72	28-69	3#, po, tid	placebo	severity of radiation enteritis

Abbreviation: NA, not available; po, oral administration; tid, three times a week; QOL, quality of life; PRS, Patient-Reported Symptoms;

**Table 2:** methodological quality evaluation of included studies

Studies	Randomization	Allocation	Blinding	Selective reporting	Other bias
Kozelsky, 2003	Y	N	Y	N	NR
Yang, 2004	Y	N	N	N	NR
Zhou, 2007	Y	N	N	N	NR
Rao, 2009	Y	N	N	N	NR
Qi, 2011	Y	N	N	N	NR
Wu, 2011	Y	N	Y	N	NR
Zhang, 2012	Y	N	N	N	NR
Gu, 2012	Y	N	N	N	NR
Zhao, 2013	Y	N	N	N	NR
Vidal-Casariego, 2014	Y	N	Y	N	NR
Feng, 2014	Y	N	N	N	NR
Chi, 2015	Y	N	N	N	NR
Chen, 2015	Y	N	N	N	NR

Abbreviation: Y, yes; N, no; NA, not available; NR, not reported

Quality evaluation using the Cochrane 5.0.1 handbook was performed on all of the eligible studies for meta-analysis [25]. These trials were mainly conducted in 2 countries(China and Spain). There was a tendency towards lower risk of bias in the articles published in English compared to Chinese studies. Tumor type, duration and dose of radiotherapy were described in most of the included studies. 9 studies reported glutamine for curing acute radiation enteritis and 4 articles reported glutamine for chronic radiation enteritis. 10 of the 13 studies identified administration of glutamine as beneficial for curing and preventing radiation enteritis, and the other 3 studies showed no significant effect of glutamine on radiation enteritis.

### Results of meta-analysis

#### Efficacy of glutamine on radiation enteritis

**Figure 2:**
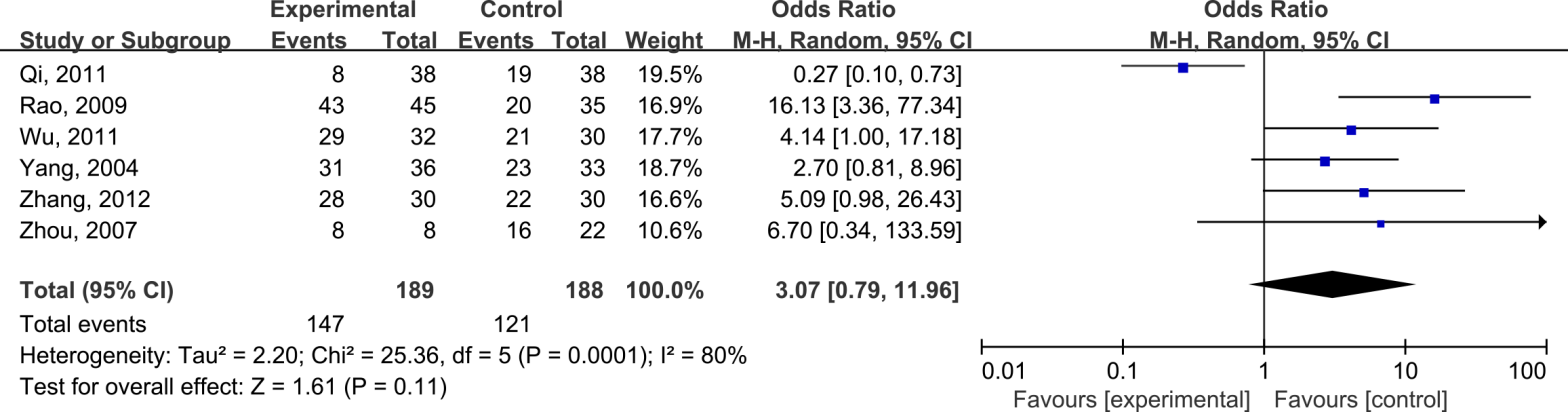
Pooled analyses on efficacy of glutamine in improving radiation enteritis

The result of the summarized estimates were shown in Figures 2. Significant heterogeneity was found among the eligible studies(*P*<0.05), so the random model was used. Overall, the combined OR for all 7 studies evaluating administration of glutamine on treatment of radiation enteritis was 3.07(95%CI: 0.79-11.96; *P*>0.05), suggesting that glutamine was beneficial for relieving radiation enteritis, however, there was no statistically significance.

**Figure 3:**
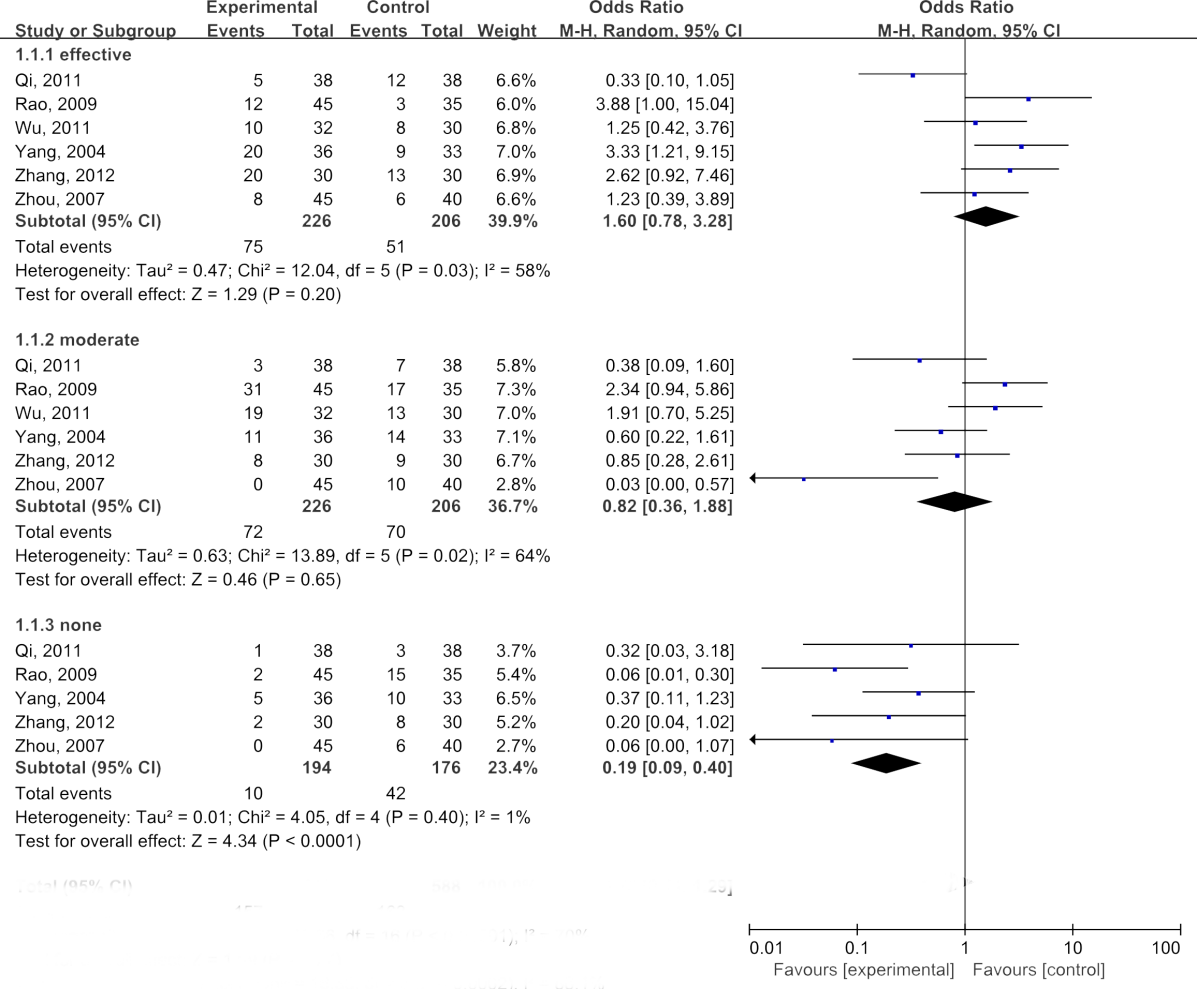
Meta-analyses of glutamine in relieving severity of radiation enteritis

Subgroup analyses were performed to evaluate the relieved degree of radiation enteritis when treated with glutamine. Three degrees of effectiveness were used as following: effective, moderate, and none effective. As illustrated by Figure 3, the combined analyses suggested that administration of glutamine could improve radiation enteritis, though there was no statistically significance(OR=1.60, 95%CI: 0.78-3.28; *P*=0.2).

#### Severity of radiation enteritis

**Figure 4:**
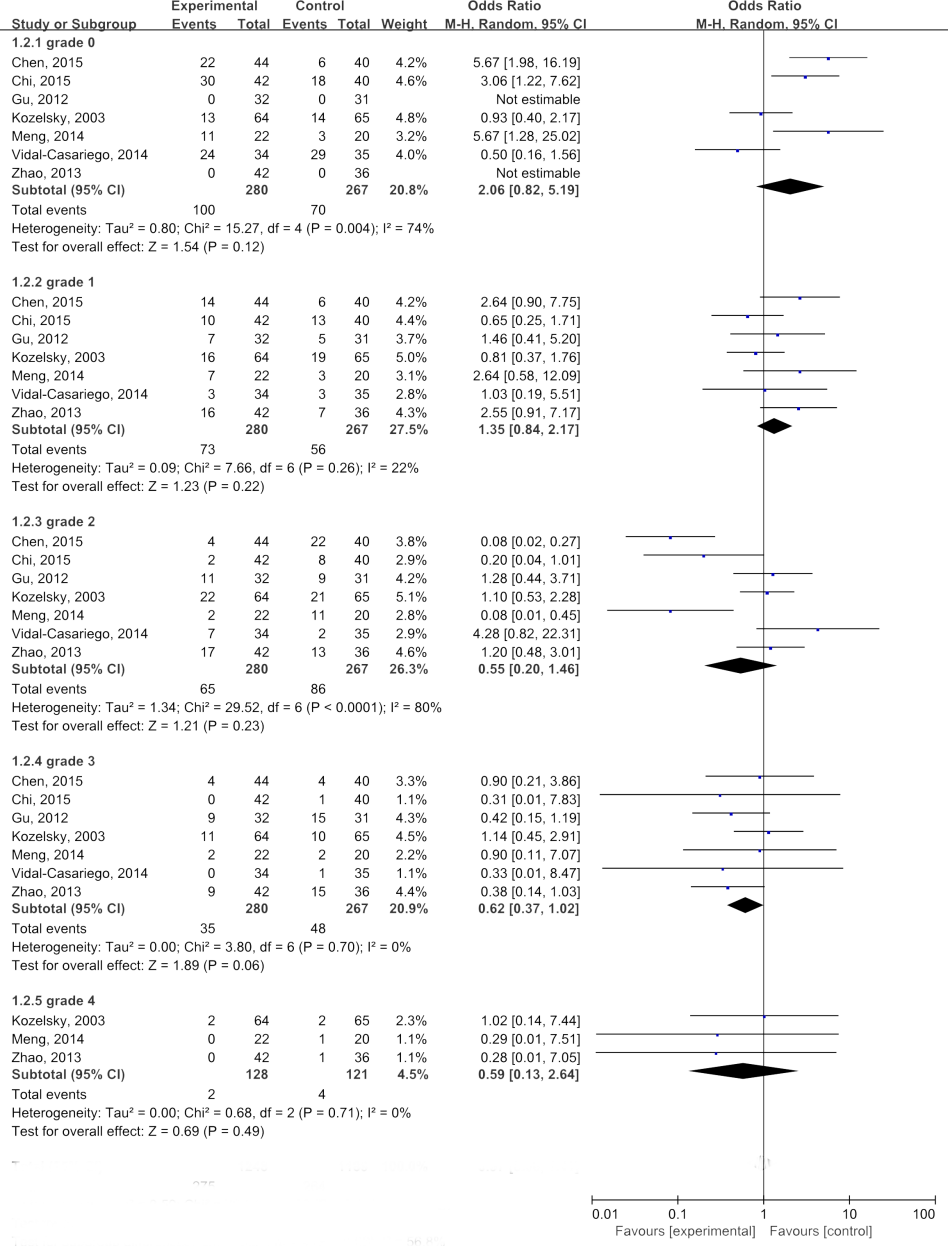
Subgroup analyses of glutamine in relieving severity of radiation enteritis

As shown in Figures 4, there were five grades of radiation enteritis, with grade 4 being the most severe one. The combined ORs for all 5 grades(from grade 0 to grade 4) of radiation enteritis in patients receiving glutamine were 2.06, 1.35, 0.55, 0.62 and 0.59, respectively. None of them was statistically significant(*P*>0.05). These results indicating that administration of glutamine failed to significantly reduce the grades of radiation enteritis.

### Symptoms of radiation enteritis

#### Tenesmus

**Figure 5:**
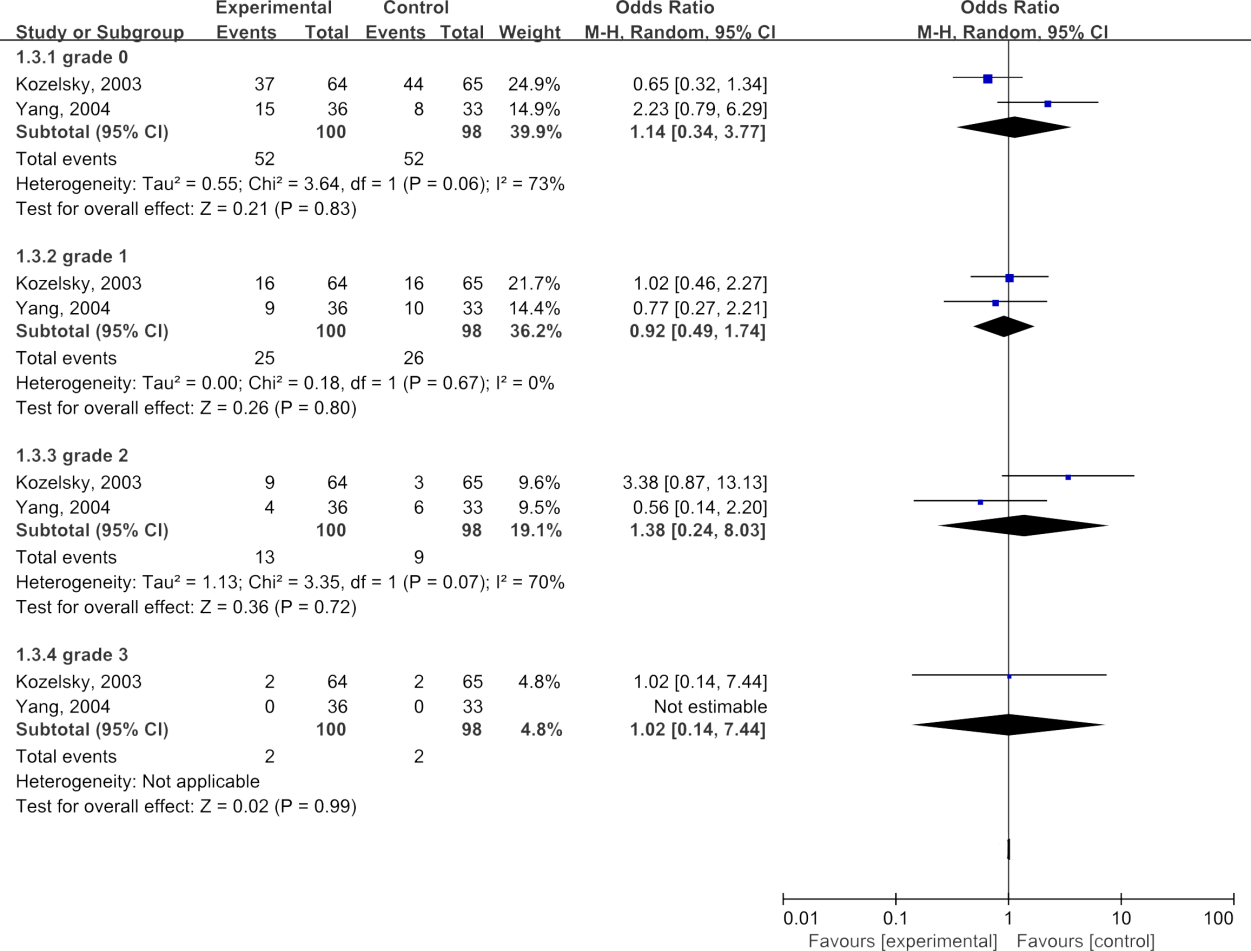
Meta-analyses of glutamine in improving degree of tenesmus

The results of the summary estimates were shown in Figures 5. Overall, the combined ORs for all studies evaluating administration of glutamine in improving grades of tenesmus were 1.14(95%CI: 0.34-3.77) for grade 0, 0.92(95%CI: 0.49-1.74) for grade 1, 1.38(95%CI: 0.24-8.03) for grade 2 and 1.02(95%CI: 0.14-7.44) for grade 3, respectively, suggesting that glutamine was not so significantly effective in treating tenesmus(*P*>0.05).

#### Abdominal cramping

**Figure 6:**
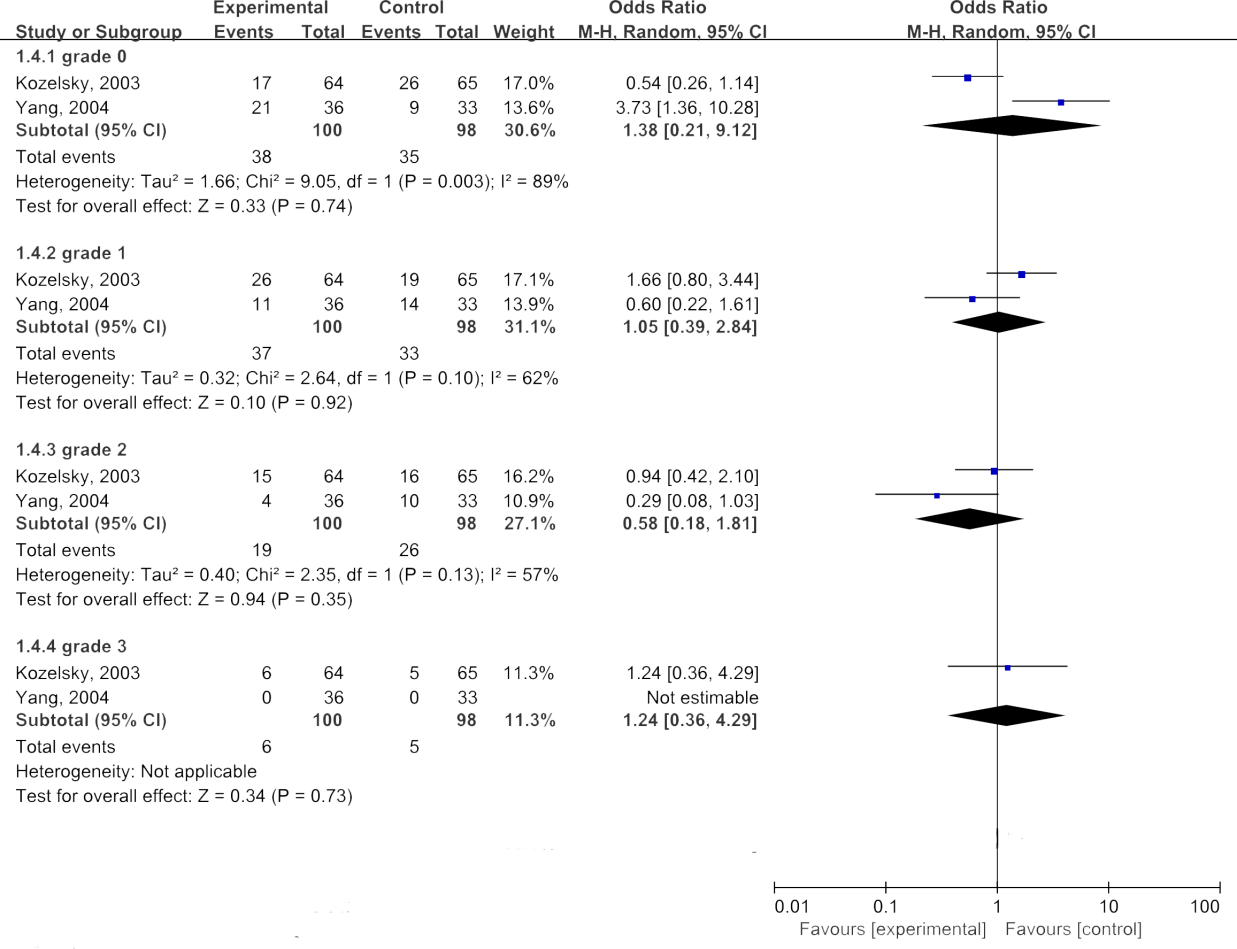
Combined analyses of glutamine in improving abdominal cramping

With regard to abdominal cramping, we also performed subgroup analyses according to the grades of abdominal cramping. The results of the summary estimates were shown in Figures 6. Overall, the combined ORs for all 2 studies evaluating administration of glutamine on reducing severity of abdominal cramping were 1.38 for grade 0, 1.05 for grade 1, 0.58 for grade 2, and 1.24 for grade 3. These findings suggested that the severity of abdominal cramping at any grade was not significantly benefited from treatment of glutamin(*P*>0.05).

#### Blood in bowel movement

**Figure 7:**
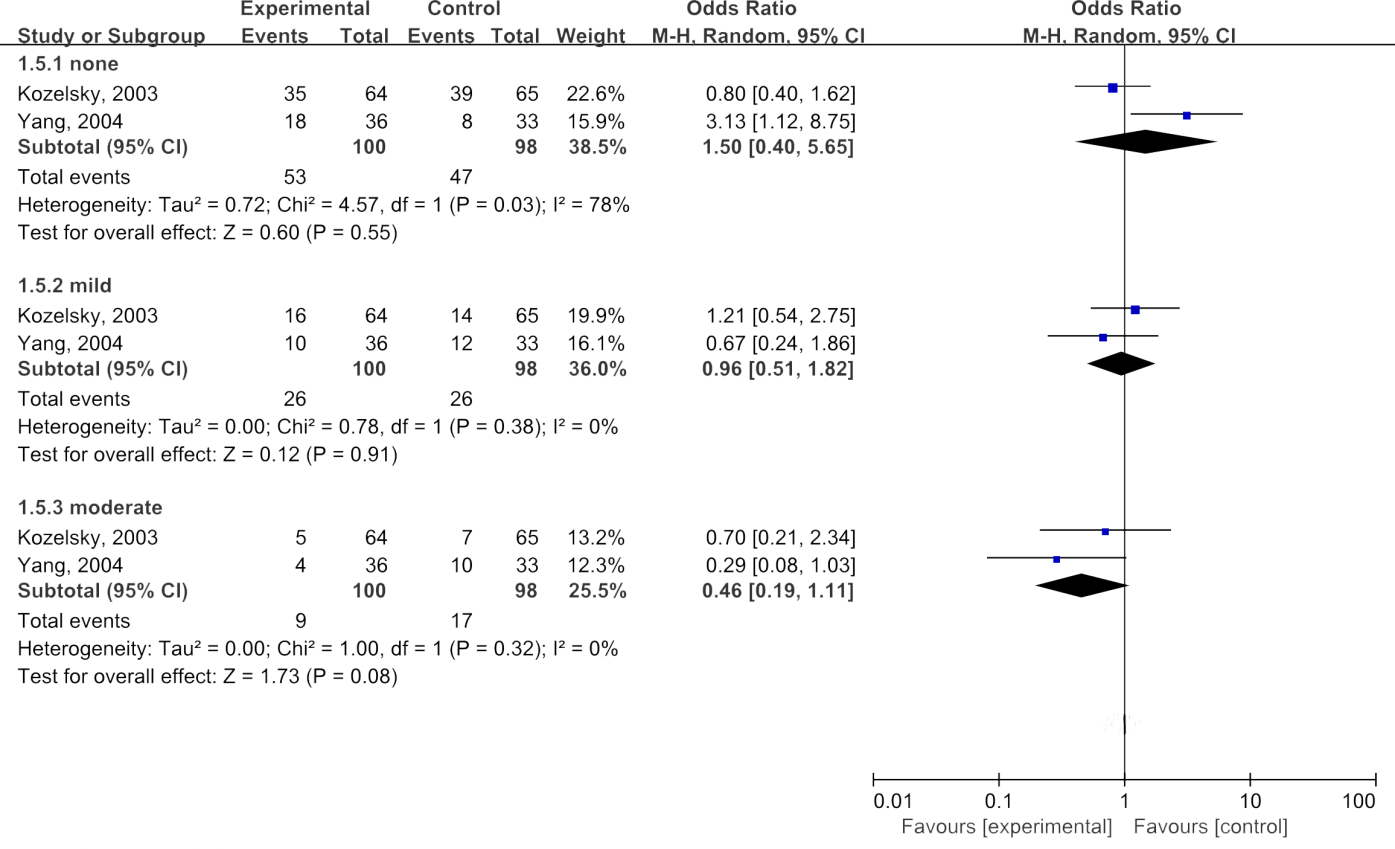
Meta-analyses of glutamine in improving blood in bowel movement

The synthesized ORs for all 2 studies evaluating administration of glutamine on reducing severity of blood in bowel movement were 1.50(95%CI: 0.40-5.65) for none symptoms of blood in bowel movement, 0.96(95%CI: 0.51-1.82) for mild, and 0.46(95%CI:0.19-1.11) for moderate, respectively(*P*>0.05 for all). These results suggested that glutamine did not significantly prevent or improve symptom of blood in bowel movement in terms of any grades(Figure 7).

#### Assessment of publication bias

**Figure 8:**
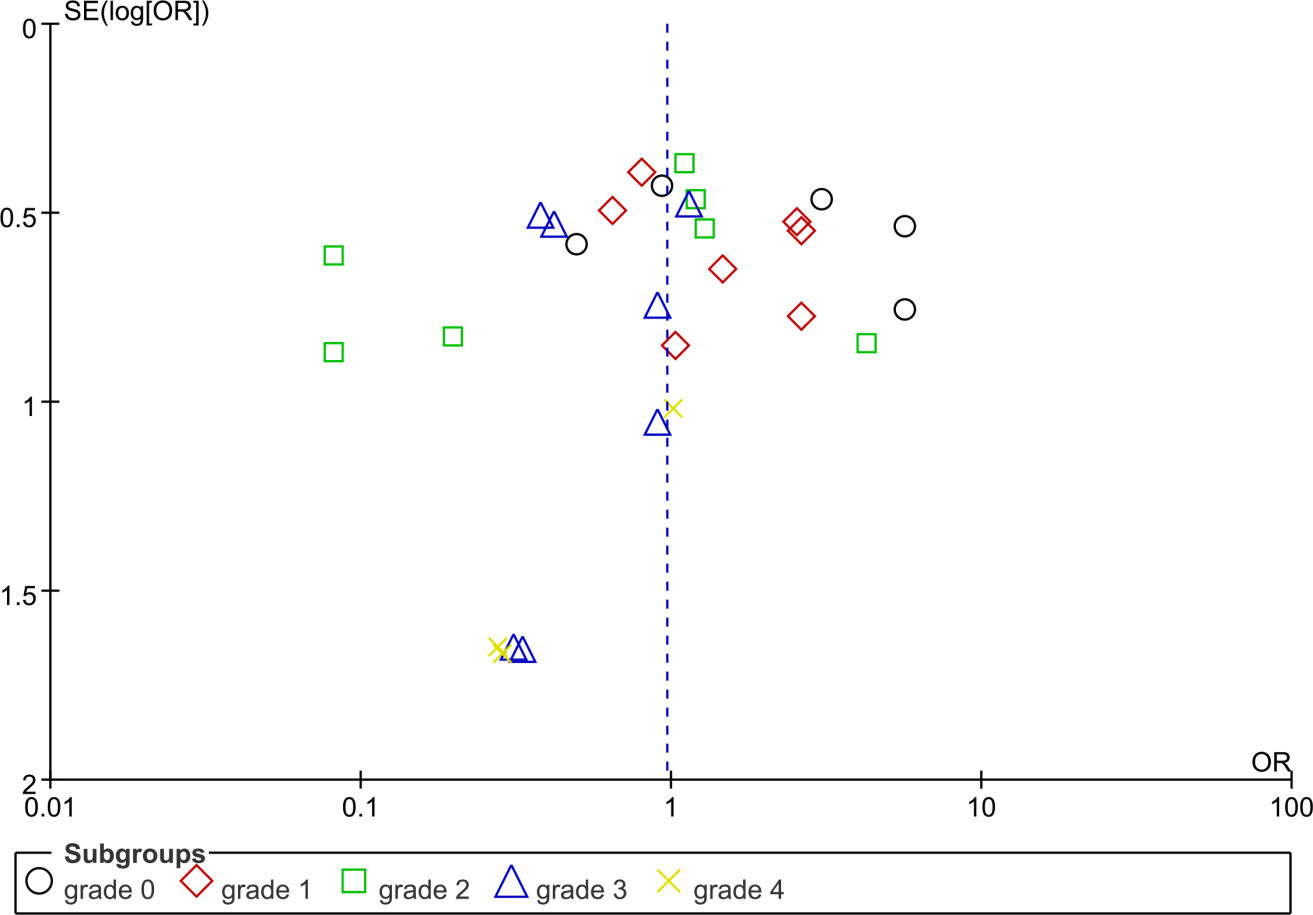
Funnel plot for detecting publication bias

We introduced Begg's funnel plot and Egger's test to evaluate the publication bias in the included references. According to the funnel plot(Figure 8), evidence of significant publication bias was found for included studies of the pooled analysis. Formal assessment of Egger's test also showed significant bias of publication in these summary analysis(*P*<0.05).

## DISCUSSION

There has been great interest in determining effective agents for curing patients with radiation enteritis. However, no popular recognized treatments have been identified through a large amount of clinical studies. Glutamine is suggested to be a powerful agent in preventing and treating radiation enteritis as proved by several animal studies and few clinical studies, but it failed to gain the same effect in some clinical trials [3, 8, 9, 11]. To gain as much information as possible, it is thought to be beneficial to evaluate the pooled effect of glutamine on radiation enteritis by using meta-analysis. In this study, we assessed the efficacy of glutamine in treating radiation enteritis and relieving symptoms of radiation enteritis. From our systematic review and meta-analysis, we included and assessed 13 full-text studies from published references comparing therapeutic efficacy in radiation enteritis patients receiving glutamine versus other treatments. Summary estimates suggested that administration of glutamine correlated to reduced incidence of radiation enteritis, higher response rate as well as improved symptom, though there were no statistically significances.

In a previously published meta-analysis [26], the authors assessed nutritional interventions for reducing gastrointestinal toxicity in patients undergoing radical pelvic radiotherapy, they referred nutritional support interventions to standard oral nutrition supplements including amino acid-based supplement and those where novel substrates have been added such as glutamine. They found that there had been benefits demonstrated with dietary modification during pelvic radiotherapy. However, they were unable to determine the effect of elemental diet as data were not available [26]. Our data provided evidence to support the beneficial effect of supplements of glutamine in curing radiation enteritis and were consistent with the results of the clinical studies published in China, however, it lost its significance. Searching, selecting, and quality assessment were conducted independently by two reviewers. Our study also checked heterogeneity and potential bias according to the published guidelines. When removing all of the trials conducted in China, the positive effect of glutamine on radiation enteritis disappeared. This could be explained by the administration of glutamine varied between China and other countries. Most of the studies published in English did not show a positive role of glutamine on radiation enteritis but not chemotherapy induced enteritis [2, 6, 10]. Membrive et. al. [2] conducted a study to determine the effect of glutamine in the prevention of induced enteritis after pelvic radiotherapy, and they found that incidence of diarrhoea observed was similar to published series in which glutamine was not administrated. They concluded that administration of glutamine to patients during pelvic radiotherapy did not appear to reduce the occurrence of enteritis [2]. This was similar with the results of some of the included trials in our meta-analysis.

As glutamine has been a powerful agent in helping recovery from tissue damages, there were several meta-analysis evaluating the therapeutic efficacy of glutamine in other inflammatory diseases, such as Crohn's disease, critical illness, oral mucositis, and respiratory disease [27–30]. Some of these studies concluded that glutamine could reduce the risk and severity of inflammation compared to either placebo or best supportive care or no treatment [28, 29]. For Crohn's disease, there was insufficient data to confirm the efficacy and safety of glutamine for induction of remission [27]. With regards to radiation-induced severe oral mucositis, a meta-analysis revealed that glutamine significantly reduced the risk and severity of oral mucositis during radiotherapy or chemotherapy [29]. These different outcomes of glutamine intervention may be due to the site, dose, duration of radiotherapy, and types of cancer and normal tissue, and other associated factors.

There are several limitations in the present meta-analysis. Firstly, though there is no significant heterogeneity among included trials, it is worthy to note that the unpublished negative results were not included in the analysis, increasing the risk of false positive results. Secondary, some of the included studies were low quality with small number of participants and different criteria of efficacy. This may reduce the reliance of the summarized estimates. Thirdly, although all of the included studies were randomized controlled trials, some of them did not mention the randomization methods, blinding, allocation concealment as well as missing data, resulting in high risk of publication and reporting bias. Forth, we planned to perform sub-group analysis according to age, treatment duration, severity of radiation enteritis and other factors. However, it was failed because of insufficient available data and may over-estimate the true efficacy of glutamine. Though these mentioned shortages may lower the reliance of our conclusion, our study systematically assessed the efficacy of glutamine in preventing and treating radiation enteritis. Meanwhile, more elegant clinical trials are welcomed to further determine the effect of glutamine on radiation enteritis in the future.

In conclusion, our meta-analysis explored the efficacy of glutamine in preventing and treating radiation enteritis and relieving symptoms of radiation enteritis. As demonstrated by our study, administration of glutamine was associated with improved clinical efficacy and relieve of symptoms, and no significant heterogeneity was found between included trials. In order to strengthen the findings, more well-designed, randomized clinical trials are required to further estimate the efficacy of glutamine on radiation enteritis.

## MATERIALS AND METHODS

### Protocol and registration

This review was performed following the instruction of PRISMA. This study had no official registration information.

### Eligibility criteria

Randomized clinical trials reporting protective efficacy of glutamine versus placebo in preventing occurrence of radiation enteritis or curative efficacy of glutamine versus placebo in cancer patients with radiation enteritis after receiving pelvic and/or abdominal radiotherapy were included. Language limited to English and/or Chinese. The primary endpoint was overall response rate, calculated by the following formula: overall response rate(%)=(total case - no response case)/ total case×100%. No response was defined as symptoms of radiation enteritis were not relieved or even exacerbated. The secondary endpoint was symptom score. Reports of animal studies were not included. Studies with reduplication, incomplete data reporting, data missing, statistical error and/or efficacy assessed without using standard criteria were also not included.

### Information sources and search

Embase, Pubmed, The Cochrane Library, CNKI (China National Knowledge Infrastructure) and other online electronic databases were used to search for eligible studies with date limited to April, 2016. The systematic search was performed by using the following terms: “glutamine”, “cancer”, “radiation enteritis”, “rectitis”, “enteritis”, “proctitis”, “radiotherapy”, “radiation”, and “efficacy”. The terms were differently combined for different database. For articles without full-text, we contacted authors to obtain detailed data. We also used professional scholar search to identify additional studies. The references cited in published papers were also taken into consideration.

### Study selection

The search was performed by two authors(De-dong Cao and Min Xu), independently. The titles and abstracts of the identified studies were reviewed, and the potential eligible articles were included for the full-text review. For trials with controversial comments, discussion was applied or third reviewer was introduced to decide whether it should be included or not.

### Data extraction and quality assessment

Data extraction and quality assessment were performed by two reviewers(De-dong Cao and Hui-lin Xu), independently. Baseline characteristics of included trials including title, authors, year of publication, journal name, cases, sex, age, interventions for treatment group and control group, definition of efficacy, primary and secondary endpoints were extracted. The obtained data were confirmed by the two authors. If any data mentioned above was not available(NA), it was recorded as NA. As most of the included articles were randomized controlled trials, quality of included studies, referring to randomization, blinding, complete reporting, and other bias, was assessed following the quality control methods in the Cochrane 5.0.1 handbook.

### Statistical analysis and synthesis of results

The meta-analysis of included studies was performed using the RevMan 5.3 software provided by the Cochrane collaboration. Eligible studies with efficacy data were included, and relative risk(RR) and its 95% confidence intervals(95%CI) with regarding to overall response rate(ORR) and symptom score were used for the overall analysis. If the ORR and other endpoints could not be directly extracted from the primary articles, it would be calculated manually. The χ2 test was introduced and inconsistency value(I^2^) was calculated to detect heterogeneity between eligible trials according to the Peto's method. The fixed effect model was used when the heterogeneity between studies was moderate(*P*<0.1, 30%<I^2^<50%) or not important(*P*<0.1, 0%<I^2^<40%), whereas the random effect model or sub-group analysis was applied if the heterogeneity was significant(*P*<0.1, I^2^>50%). Here, we define the heterogeneity was moderate with the I^2^ ranged from 30% to 50%, but not 60% mentioned by the handbook 5.1.0 [25]. The pooled effect of glutamine on preventing or curing radiation enteritis was defined to be statistically significant if the *P*<0.05.
